# A Qualitative Exploration of Causes of Depression among Persons Living with HIV Receiving Antiretroviral Therapy in Uganda: Implications for Policy

**DOI:** 10.1155/2023/1986908

**Published:** 2023-01-17

**Authors:** Rwamahe Rutakumwa, Christine Tusiime, Richard Stephen Mpango, Leticia Kyohangirwe, Pontiano Kaleebu, Vikram Patel, Eugene Kinyanda

**Affiliations:** ^1^Medical Research Council/Uganda Virus Research Institute & London School of Hygiene and Tropical Medicine Uganda Research Unit, Plot 50-59 Nakiwogo Road, P.O. Box 49 Entebbe, Uganda; ^2^Butabika National Referral Mental Hospital, Old Port Bell Road, P.O. Box 7017 Kampala, Uganda; ^3^Department of Global Health and Social Medicine, Harvard Medical School, Boston, MA 02115, USA; ^4^Department of Psychiatry, Makerere University, P.O. Box 7072 Kampala, Uganda

## Abstract

**Introduction:**

Depression is the fourth leading cause of the global disease burden and worsens the outcome of comorbidities including HIV/AIDS. Depression is particularly problematic among persons living with HIV in sub-Saharan Africa where scarcity of cost-effective interventions is compounded by inadequate understanding of the disease. We examine risk factors for depression among persons living with HIV undergoing antiretroviral treatment in Uganda and discuss policy implications.

**Methods:**

A qualitative study using a narrative approach was conducted, the formative phase of a large study to develop a model for integrating depression management into routine HIV care in Uganda. Participants were purposively sampled at four public health facilities in Mpigi District. In-depth interviews were conducted with four clinicians, three supervisors, and 11 persons living with HIV and suffering from depression, as were three focus group discussions with lay health workers. Exit interviews were conducted with 17 persons living with HIV who completed/interrupted depression treatment but had not been interviewed. Only data collected from persons living with HIV and lay health workers were analysed for the purpose of this paper. A narrative thematic approach was used in data analysis. *Findings*. There were several pathways through which lack of family social support reportedly led to depression: worries about disclosure in discordant relationships, false perceptions of social support, stigmatisation and discrimination, and domestic violence. Economic/poverty and other causes were identified, but their role was less significant or moderated by family social support.

**Conclusion:**

Family social support plays a dominant role—both directly and indirectly—in influencing depression risk. We propose the mainstreaming of formal psychosocial support and a shift from individual to family-focused counselling that targets both persons living with HIV and their family.

## 1. Introduction

Depression is now the fourth leading cause of the global disease burden [1], affecting an estimated 350 million people globally [2], and is the leading cause of disability worldwide [1, 3, 4]. Depression continues to be a neglected mental health disorder in sub-Saharan Africa [5], where data on the prevalence and scope of the disease as well as the resources to address it are scant [6]. Depression significantly affects the quality of life of patients [2], in many cases exacerbating the outcome of other physical health problems [7, 8], and is also a risk factor for suicide [2, 6]. Among persons living with HIV/AIDS, depression has also been associated with other negative clinical and behavioural outcomes [5, 9], including faster HIV disease progression [10] poor adherence to antiretroviral therapy (ART) [11, 12], poor linkage to care [13], treatment failure and drug resistance [14], and risky sexual behaviour [9].

In sub-Saharan Africa, depression is not sufficiently understood. Indeed, our review of literature, for example, points to persistent gaps in the understanding of psychosocial and environmental risk factors for depression [15]. Consistent with this observation, we note in our review that while there is general concurrence around several risk factors, including age, marital status, living alone, poor medication adherence, stigma, income, and poor social support [16–18], not as much attention has been paid to the apparently important family environment/family social support as a risk factor for depression among persons living with HIV and AIDS (PLWHA). Yet, social support has been associated with better health outcomes especially for PLWHA [19], more so those at risk of depression [20]. It is therefore important to explore not just service providers' but also patients' perspectives/beliefs about depression, including its causes, in order to design effective public health interventions for both prevention and treatment of the health condition.

Our purpose in this paper was to examine risk factors for depression among persons living with HIV undergoing antiretroviral treatment in Uganda and to discuss implications for policy and practice.

The theoretical underpinnings of this paper revolve around the concept of “social support”—the transfer of resources from the giver to the recipient for the purpose of enhancing the wellbeing of the recipient [21]. While the literature on social support and health has featured a variety of theoretical strands, the strand we consider most pertinent to our work is the “stress and coping theory” [22]. This theory stipulates that social support acts as a stress buffer by moderating the valuation of stressful life events (in this case HIV/AIDS), resulting in the perception of such events as less threatening [22–24]. Based on the theory, this buffering effect is delivered by way of either other persons' supportive actions, which has been categorised as “received support” [25] or trusting that support will be available when needed, that is, “perceptions of social support” [25].

Cutrona and Suhr [26] have conceptualized social support using five general categories: emotional, informational, esteem, social network, and tangible support. According to the authors, emotional support refers to expressions of empathy, love and care; informational support speaks to transmission of knowledge and advice; esteem support is about compliments and validation of one's abilities and value; social network support includes messages that enhance one's sense of connection or companionship with others; and tangible support is the assistance in form of good and services [26]. While these categories of social support may not necessarily be mutually exclusive in their dispensation, their differential impact is worth noting. In multiple studies conducted in various settings [27–29], for example, emotional support was found to be the most useful of the different categories in enhancing mental health. While none of these studies was conducted in a sub-Saharan African setting, observations from our current and earlier studies undertaken in the Ugandan setting point to the critical role of especially emotional support in improving health outcomes for persons living with HIV/AIDS and depression comorbidity.

Drawing from these studies and our own observations from the current study, we therefore view emotional support—exhibited in empathy, love, and care—as the most important for understanding the onset and experience of depression among persons living with HIV in our study. Accordingly, we propose that absence of emotional support, either perceived or received, accounts in large part for the incidence of depression among persons living with HIV and AIDS, and that the absence of such support, especially within the family, has both a direct causal relationship with depression and an indirect one by moderating the impact of poverty and other factors such as nondisclosure and withdrawal [30, 31].

We draw on these theoretical perspectives to interpret and discuss our findings and to suggest contextually grounded strategies for addressing depression among persons living with HIV and AIDS.

## 2. Methods

### 2.1. Design and Study Setting

We conducted a qualitative exploratory study, using a narrative approach in which the inquiry centres around narratives of human experience and produces data in narrative form [32]. This narrative approach has been found to be relevant when exploring individuals' lives as told through their own stories, particularly illness experiences [33]. Accordingly, our inquiry sought to generate narratives in the form stories or oral histories from PLWHA about their illness experiences, social support systems, and causes of depression among PLWHA.

This qualitative study was part of the formative phase of the broader HIV + D intervention. The general objective of the HIV + D intervention was to develop and evaluate a model for integrating the management of depression into routine HIV care in Uganda. The formative phase had several specific objectives, including one that initially inspired this qualitative study, namely, to evaluate the acceptability and feasibility of an intervention that will integrate the management of depression into routine HIV care in Uganda (the HIV + D intervention).

The formative phase of the HIV + D intervention was undertaken at four public health care facilities (PHCFs) in Mpigi District in central Uganda. The District operates 19 PHCFs with varying levels of care, graded as health centre (HC) II, HCIII, HCIV, and a district hospital. The health facilities run HIV clinics weekly. These clinics are staffed with general health workers, counsellors, and lay health workers who do HIV pre- and postcounselling, ART, and adherence counselling. It is worth noting that the lay health workers were themselves PLWHA and had recovered from depression and had undergone counselling training. The HCIV (health subdistrict) serves as the referral point for the lower health centres and may in turn refer to the district hospital. Health centres II and III have no specialised mental health care, and individuals identified as suffering from a mental illness are referred to HCIV or the district hospital with only a handful of psychiatric nurses who largely work in nonmental health clinics, leaving much of the district with no access to psychiatric care. Depressed PLWHAs are only identified if their situation is severe enough to cause a high viral load, in which case they are given antidepressants or referred to counsellors with training in HIV adherence.

The four selected PHCFs, where the HIV + D intervention was undertaken, represented three levels of the Ugandan health care system: Butoolo and Buwama, with level three health centres (HCIII); Mpigi, with level four health centres; and Nkozi, with a hospital level health facility.

### 2.2. Study Participants

The formative phase aimed at developing and piloting the HIV + D intervention. During this phase, different studies were undertaken, pertinently including the qualitative study on which this paper was based. These studies jointly drew from a purposive sample of a range of participants including clinicians, supervisors, lay health workers, and PLWHA receiving ART and suffering from depression. The participants were approached in person by the research team at the public health care facilities where they provided/received care.

### 2.3. Data Collection Procedures

Qualitative data were collected from PLWHA and lay health workers and from clinicians and supervisors, although the latter two categories of participants were not included in the analysis for this paper. Using semistructured interview topic guides, three in-depth interviews were conducted with each of 11 PLWHA who were on depression treatment during their health care facility visits. Additionally, three focus group discussions were held with lay health workers. The in-depth interviews and focus group discussions covered a range of topics as highlighted in Table 1 below. In addition, when exploring PLWHA's knowledge of depression—one of the main research questions on the interview guide—the role of family in influencing the risk of depression among PLWHA consistently came up unsolicited during the interviews. It is on this note that we also set out to probe and obtain detailed narratives from PLWHA and lay health workers on the illness experiences, social support systems, and causes of depression among PLWHA. These interviews and group discussions were conducted at different time points over a period of six months—at baseline (month 0), midline (month 3), and end line (month 6). Besides, exit interviews were conducted with a subset of PLWHA who had not been interviewed, including (i) 11 who had been recategorised as adherent in their depression treatment on the pilot intervention study and (ii) six PLWHA, recategorised as nonadherent on account of missing at least two psychotherapy sessions. The exit interviews initially had been aimed at exploring experiences and reasons for adherence/nonadherence.

Across all the different categories of participants in this qualitative study, sufficiency of the number of participants interviewed was determined when additional interviews elicited no new information, that is to say, when we achieved data saturation [34].

All interviews and focus group discussions were conducted/moderated by research assistants with a psychiatric nursing background and who had undergone a rigorous training in qualitative interviewing. The interviews were audio-taped and transcribed verbatim. Interviews and focus group discussions with patients and lay health workers were conducted/moderated and transcribed in the local language (Luganda). These were then translated to English by the interviewers/moderators who conducted the respective interview/focus group discussion. Interviews with clinicians and supervisors were conducted and transcribed in English. All the interviews and focus group discussions were conducted at facilities where the participants received/provided care. The interviews on average lasted 45 to 60 minutes while focus group discussions lasted one to two hours.

### 2.4. Data Analysis

For the purpose of this paper, we analysed data from PLWHA and lay health workers who themselves were living with HIV, specifically examining their detailed narratives on their illness experiences, social support systems, and causes of depression among PLWHA. Two expert qualitative researchers conducted the data analysis in different time periods. The first researcher conducted a data-driven analysis aimed at an initial in-depth exploration of the data. Building on the output from this broader analysis, the second researcher conducted an analysis focusing on the overarching theme of this paper—causes of depression among PLWHA. The outputs of the analysis from the two researchers were reviewed by the study team and found to be generally consistent and accurate.

We used a “narrative thematic analysis” approach, one of several approaches of narrative analysis, whose basic focus is content within the text [35]. The goal was to gain an understanding of patterns of meanings from the data on the experiences of HIV-related illness, social support systems, and causes of depression among persons living with HIV. The analysis followed an iterative process initially involving reading all the interview transcripts (preliminary analysis) in order to achieve familiarity with the data, after which a more in-depth analysis of individual transcripts was conducted in which data were broken down into multiple units of meaning with respect to the subject of the investigation (causes of depression). These units of meaning were then linked together to form clusters (subthemes) from which key themes were eventually abstracted.

Data were coded with the help of a thematic framework initially developed from the preliminary analysis and concepts borrowed from our theoretical framework and refined as more data were analysed. Using MS Excel, the key themes and subthemes in the thematic framework were classified on a matrix, which was then used to capture relevant pieces of data that illustrated the themes. Our analysis yielded the following themes and subthemes: (1) family-related causes, with subthemes including worries about disclosure in a discordant relationship, false perceptions of social support, stigmatisation and discrimination, and domestic violence; (2) economic causes; and (3) other causes.

### 2.5. Validity and Reliability

In qualitative research, validity refers to accuracy of the findings, while reliability is about consistency of the analytical procedures across researchers and datasets [36]. Creswell [37] has recommended the use of at least two strategies for the findings of a given study to be considered valid and reliable. In our case, our strategy for promoting internal validity was in ensuring that participant recruitment for interviews continued until data saturation was achieved. As well, we promoted external validity (transferability)—the degree to which the findings from qualitative research are generalizable or transferable to other contexts—by providing a “thick description” [34, 38] or detailed account of our methodological procedures, participants, and findings. With thick description, we hoped to enable the reader evaluate the extent to which our inferences and conclusions are transferable in different contexts and populations. On the other hand, our strategy for promoting reliability was the detailed, step-by step documentation of the data collection and analytical process—creating an audit trail—so other researchers are able to make informed judgments about the soundness of the process and to replicate these when necessary.

### 2.6. Ethical Considerations

Ethics approval for this study was obtained from the Science and Ethics Committee of the Uganda Virus Research Institute Research Ethics Committee, the Science and Ethical Committee of the London School of Hygiene and Tropical Medicine, and the Uganda National Council for Science and Technology.

## 3. Results

### 3.1. Family-Related Causes of Depression: Absence of Emotional (Social) Support within Family

In a study context where the traditional, informal, social support system still predominates as a source of social care, family relationships might ordinarily have been expected to deliver protective effects against depression among persons living with HIV. Yet, results of our data analysis show that the family was the major and most frequently cited source of depression among PLWHA participating in our study. Given this relatively high emphasis that PLWHA placed on the role of family relationships, the importance of these as a risk factor for depression appeared to supersede even that of poverty, in spite of the widespread economic insecurity in the study area. We present below the different pathways through which the family environment, specifically absence of emotional support, led to depression among persons living with HIV. These pathways included worries about disclosure in a discordant relationship, false perceptions of social support, stigmatization and discrimination, and domestic violence.

#### 3.1.1. Worries about Disclosure in a Discordant Relationship

For some of the participating persons living with HIV, specifically those in a discordant relationship, depression was the ultimate outcome of fear of disclosure to a spouse where there was a perception that they would not be supportive, which resulted in withdrawal from engagement with the spouse on matters related to their HIV status. This perceived lack of support led to constant worries on the part of the spouse living with HIV about how they would disclose their status to the partner without triggering the much dreaded scenario of a break-up of the relationship. These worries were particularly complicated by the imperative of daily antiretroviral therapy (ART), which made unintended disclosure a daily risk. In navigating this challenge, seropositive spouses habitually performed a near-futile, day-to-day balancing act between two competing necessities: ensuring, on the one hand, that their seronegative spouse does not get to know that they are living with HIV and, on the other hand, adherence to their ART regimen. It is the emotional exhaustion arising from having to confront this challenge on a daily basis that culminated in depression.

“For my case the cause of depression was that I was in a discordant relationship in which I was living with HIV…. So it took her some time before knowing that I was sick. That kept me worried about how I would break such disturbing news to her. I would always wonder in case she got to know what her reaction would be. [My worries were also based on] the statements that she used to make, that ‘in case we test with my husband and I am found negative when he is positive, I will just divorce him'. So that made my disclosure hard as I feared what would happen after telling her. Sometimes I would think she might abandon me with these young children. That would worry me a lot, causing sleeplessness…. It was like I was in prison.” (Nkozi, Psychotherapy 3, Baseline Interview, Male).

In other instances, the perceived lack of emotional support and subsequent disposition not to disclose pushed the infected spouse towards the difficult choice of abandoning ART. However, this itself did not offer relief as they still had to worry about issues of ART adherence, which sometimes led to depression.

“I think even being in a discordant relationship [can cause depression]. For example, some women after realizing that their husbands are negative they also stop swallowing drugs thinking that if the husband sees her taking the ART she will be abandoned. So they worry about how to take their drugs and become depressed.” (Buwama, Psychotherapy 2, Baseline Interview, Female).

#### 3.1.2. False Perceptions of Social Support

Based on PLWHAs' narratives on the psychosocial complications of an HIV infection, it was clear that the inability to draw empathy from blood relatives was hard for the family member living with HIV to appreciate. Drawing on Edwards and colleagues' [25] theoretical perspectives as highlighted earlier, we view this as a case of false perceptions of social support because the support that the seropositive family member had taken for granted turned out to be unforthcoming. The implication of such false perceptions for the risk of depression was apparent in cases where those who turned out to be unsupportive were immediate family members.

“My own mother, the one I would have at least shared my problems with, showed me that she was not interested in knowing what I was going through. I really got psychologically perturbed due to the fact that my real mother did not mind about me, and yet she would have been the one to comfort me.” (Butoolo, Antidepressants, Adherent, Exit Interview, Female).

This sentiment was echoed by a lay health worker while sharing perspectives from a session with another person living with HIV.

“Someone tells you: ‘I had a secret but had no one to tell it to. People at home do not care what happens in my life. But when I am here and I tell you about my problems, I feel much better'. And you see them very happy just because of that.” (Lay health worker, Focus Group Discussion, Baseline).

Relatedly, our findings show that this lack of empathy from the taken-for-granted sources was another cause of withdrawal by persons living with HIV from active engagement with family. In such cases, the patients opted to keep to themselves not just their thoughts and concerns but their depression treatment programme as well. We observed that withdrawal was in itself a risk factor for depression insofar as it effectively blocked potential support that might have helped in the management of stress and preempted depression. This perspective was shared by lay health workers during a group discussion, in which they reflected on the social challenges of persons living with HIV based on the lay health workers' own experiences.

“So for all the symptoms the other counsellors have been talking about, people live with them silently and most of the time have nobody they can talk to – not even their family members. They keep in that state silently. Sometimes they may have long thought about committing suicide but fear that ‘maybe if I talk to my wife, the response will be negative', so they keep hurting silently in their homes because nobody asks them about it. So their lives keep getting worse.” (Lay health worker, Focus Group Discussion, Baseline).

The frequency of such lack of support for family members living with HIV was highlighted during a subsequent group discussion with lay health workers, in which the role of gender was also notably indicated.

“Most of our female clients had family problems and as such in most cases we would refer them to NGOs to get help.” (Lay health worker, Focus Group Discussion, Endline).

Lack of empathy from family members also took the dimension of insensitive advice. This perspective emerged from group discussions with lay health workers, who cited instances in which family members, with little regard for the sensitivity of an HIV diagnosis, have called on the person living with HIV to essentially make preparations for their imminent death because, in their opinion, this was an inescapable outcome of an HIV infection. We found that such advice encouraged maladaptive coping behaviour on the part of the family member living with HIV, sometimes leading to a sense of hopelessness and depression.

“You know depression comes in very fast… there are those who test and are told that they are living with HIV and when they tell family members, most of them are not aware of benefits of treatment and discourage the patient, ‘you have HIV, you are finished'. They even begin asking them the plan of where they are to leave their children, and all this causes the person to lose hope. Some members tell them they will take drugs for two months and die. They begin to remind them of all the people that have died of HIV. The person feels hopeless and eventually begins to sell all their property just like our colleague told us [one of the colleagues sold off all is property.” (Lay health worker, Focus Group Discussion, Baseline).

#### 3.1.3. Stigmatization and Discrimination

Our findings show that absence of social support within the family has also been exhibited in stigmatization and discrimination of the family member living with HIV. We do not necessarily imply that persons living with HIV encountered this challenge only within the family environment. Indeed, there were instances in which stigmatization and discrimination were encountered at the wider societal level.

“When people know that you are living with HIV, they refer to you as ‘omulambo ogutambula nga guyimiridde' meaning a walking dead body. So that causes a lot of depression.” (Nkozi, Psychotherapy 2, Baseline Interview, Female).

“I think discrimination contributes a lot to poverty because someone is denied a job because of their HIV status that causes them to be poor which causes them to be depressed.” (Buwama, Psychotherapy 2, Baseline Interview, Female).

However, contrary to what might be expected, cases of stigmatization and discrimination more often were reported to have occurred within the family environment than at the wider societal level. This observation was made based on the perspectives of those living with HIV and later reinforced during group discussions with lay health workers as they shared experiences from their one-on-one sessions with the patients.

“The [mental] state in which I was caused me to be put on second line ART because I was depressed a lot when I was still staying at home…. I was going through tough times, I had stigma and as such I could not take my medication very well, so I was switched to second line treatment.” (Buwama, Psychotherapy 1, Baseline Interview, Female).

These patients' perspectives were echoed by lay health workers, as one focus group participant noted

“Many people say they are segregated at their homes and are insulted that they have HIV. They feel they are HIV positive and are not worthy to be part of the family members.”

#### 3.1.4. Domestic Violence

Absence of an emotionally supportive, caring environment within family was additionally evident in domestic violence. This normally occurred in the context of spousal relationships, in which a male typically was the abuser. The violence was exhibited in different forms including recurrent physical abuse, denial of shelter and, in some extreme cases, death threats, which often resulted in depression. While lamenting about her frequent experiences of physical abuse and lack of empathy and understanding from the male spouse, one PLWHA also suggested the need for family-oriented counselling programmes rather than the existing individual-centred programmes.

“What I was expecting in counselling was that even my man would be called and counselled about the way he was handling me – molesting me all the time. Because the man knows very well that he is the one who infected me yet at the same time, he is the one molesting me. If he was not molesting me, I would have not developed those thoughts [wanting to commit suicide] because I did not have any problem. I wish my man had been invited so that he is sensitized and counselled about depression…. He [should] understand what wrongs he is doing and I also understand the wrongs I do, so that he knows that my [depressive] situation is due to the wrong things he does to me.” (Mpigi, Psychotherapy, Non-adherent 2, Exit Interview, Female).

For another PLWHA, the violence was in the form denial of shelter.

“My husband behaves badly towards me, he abuses me so much and chases me away from home and he treats me as if I am someone who cannot get anywhere to go or stay. Sometimes he locks me outside the house.” (Mpigi, Psychotherapy & Antidepressant, Baseline, Female).

### 3.2. Economic Causes of Depression (Poverty)

Aside from family-related causes, poverty emerged as another important cause of depression among persons living with HIV. In some cases, PLWHAs suggested a direct causal relationship between poverty and depression, whereby poverty was perceived as being entirely responsible for the depression that the person living with HIV was experiencing. This perspective emerged during both interviews with those living with HIV and group discussions with lay health workers.

“I thought that how I wish I could die because I was suffering a lot. My children had been chased away from school, I could not afford what to eat so I kept on thinking about suicide.” (Buwama, Antidepressants 2, Baseline Interview, Female).

“Every person gives you a different cause but the most common cause I have observed is poverty, lack of food. Because when one has no income, they cannot have what to eat and school fees for their children. For most of the people I ask about what causes their depression they say that every time they think about their children in addition to lack of food, the thoughts increase.” (Lay health worker, Focus Group Discussion, Baseline).

However, while our preliminary analysis had indicated poverty as the predominant cause of depression, it became clear following a more in-depth analysis of data that the impact of poverty as a risk factor for depression might in several such cases have been moderated by other factors, principally social support within the family. This indicated the predominance of family-related causes over poverty, an observation to which one of the persons living with HIV directly alluded.

“Poverty might not be such a big causative factor especially if the family members understand and cooperate with each other. But if you are being segregated because of poverty especially by your own parents then it can cause you to be depressed. For my case, I am segregated at home because I am poor and that causes me to be depressed.” (Nkozi, Psychotherapy 2, Baseline Interview, Female).

Indeed, there were cases of persons living with poverty who earlier during the interview dialogue had suggested that their depression was the direct outcome of poverty. However, as they delved deeper into the events leading to their depression, the role of family relationships in the onset of their mental illness became apparent. In one of these cases, a PLWHA (quoted earlier) who had cited the direct causal effect of poverty seemed to qualify such effect by underscoring the potentially moderating role of negative family relationships. Even as she recognised the importance of poverty, she appeared to be attributing her depressive condition largely to her entanglement in a distressful relationship with her violent husband who would not leave her alone despite the fact that they had separated.

“My husband used to disturb me a lot, he was poor and in fact unable to provide for my children, it even got to a point when they chased me away from where I was staying. So that caused me a lot of pressure, I would always worry a lot, I used to feel weak all the time, my appetite was poor and I was unable to sleep…. When I got some 30,000 Shillings I decided to rent [but] when I got to the house, the landlord refused to avail me space…. They told me that my husband would come and remove the doors because wherever I go [he does that]. So they refused to give me the house. I went and asked for a free house from a certain man who accepted. Since it was getting late, I started by transferring the bed, the owner of the house came and told me to leave the house. I asked him ‘what is the problem? You had authorized me to stay in the house and I had even cleaned the house and transferred some of my items there?' He told me that ‘your husband has told me that he is going to come and behead you, then I would be imprisoned'.… It was night time…. I went to my in-law who allowed me to stay in a very small grass thatched house. My husband continued to come and issue threats that he is going to burn me from that house. So they also chased me from that place, and I had nowhere to go now [participant cries]….” (Buwama, Antidepressant 2, Baseline Interview, Female).

### 3.3. Other Causes of Depression

In addition to the major causes of depression as presented above, we noted that only few persons living with HIV attributed their depression to health concerns arising from their HIV status. The fact that these concerns and associated fear of eventual death turned out not to be an important cause of depression among the persons living with HIV was remarkable. But this may be appreciated in the context of the current universal access to highly effective ART, as HIV is increasingly perceived as a manageable chronic condition rather than a sure path to imminent death. Nonetheless, our findings show that depression sometimes stemmed from constant worries about viral nonsuppression, especially if the person living with HIV was strictly adhering to ART. In response to a question as to what causes depression among those living with HIV, one patient had the following to say:

“Telling them [those living with HIV] that they are not swallowing their ART medication well yet to them they have been actually taking their medicines as prescribed, so they might think too much and ask themselves questions about what might be causing it. To some it might be because of taking alcohol.” (Buwama, Antidepressant 1, Baseline Interview, Male).

For other PLWHAs, the fear of imminent death did not just cause depression. It also compounded their depressive situation by triggering maladaptive coping. This occurred when the person living with HIV, in anticipation of imminent HIV-related death, withdrew from full engagement in income generation that would have been vital for their own and family welfare. This led to financial distress/poverty and subsequent degeneration in their mental health condition.

“When someone gets to know that they are living with HIV, even if they have a job, the person begins to think, ‘Why should I continue to work?' They do not easily accept that when they take drugs they are going to live longer. Most of the time they think that they are going to die so they do not see the need to work. Some people may continue to work because of their children but they put less effort to work and only look at working for survival. So the issue of people not recognizing the need to continue working when diagnosed with HIV, thinking that they are going to die anytime, brings about the poverty….” (Lay health worker, Focus Group Discussion, Baseline).

## 4. Discussion

We have shown in this paper that social (especially emotional) support from family members plays a significant role in influencing the incidence of depression among PLWHA, and that this role is not only direct but also indirect through moderation of other risk factors, notably poverty. Our findings build on previous extensive work [29, 39–42] highlighting the lack of social support as an important risk factor for depression. But in a context where the traditional social support system still predominates, and where family relationships might ordinarily have been expected to deliver protective effects against depression, our findings also contribute to the literature by illuminating the lack of social support within family as an overriding cause of depression among PLWHA in our study.

The significance of social support within family is further illustrated in our study by its role in moderating the impact of other key risk factors such as poverty, a finding consistent with those from work conducted in the developed world [43–45]. This study has revealed, for example, that when PLWHAs point to poverty as the cause of their depression, they may in fact be presenting only a partial picture of the complex causal dynamics culminating in their depressive condition. Relatedly, the study reveals that some risk factors—in this case poverty—may in some instances be impactful only in synergy with others such as lack of social support, although poverty continues to be largely depicted in the literature as an independent risk factor [2, 46–48]. This suggests that gaps still exist in the understanding of the risk factors but also underscores the value of thick description [38] in the recording of qualitative data for the purpose of obtaining a more valid and complete picture of participants' experiences and perspectives. Thus, our findings lend credence to those from Kim and colleagues' [49] study among adolescents living with HIV in Malawi, in which it was observed that risk factors for depression among PLWHA are yet to be adequately described especially in the sub-Saharan African sociocultural context.

Also, noteworthy was the withdrawal by persons living with HIV from active engagement with family on matters related to their HIV status. This reportedly was the outcome of the disillusionment from unfulfilled expectations of emotional support—or false “perceptions of social support” [25]—from the family members, which the person living with HIV had taken for granted. While the decision by the persons living with HIV to instead confide in nonfamily members/service providers might not have been expected in the sub-Saharan African context, it echoes findings from earlier studies conducted in the United States. For example, Kalichman and colleagues [30] found that persons living with HIV perceived friends as being more supportive than family members and, as a result, were more inclined to disclosing their HIV status to friends. Similarly, a United States study found that 85 percent of men living with HIV had disclosed their seropositive status to at least one friend, whereas only 36 percent had disclosed to family [50]. The fact that patients with depression were reported to withdraw from social contact supports the use of behavioural activation (as part of the Health Activity Program) [51] as a therapeutic intervention in this sociocultural context. Behavioural activation encourages patients with depression to reengage with society.

### 4.1. Policy and Other Implications

The findings from this study draw attention to the need for depression prevention and treatment approaches that are contextually grounded but also sensitive to the changing sociocultural norms and practices. Accordingly, we propose policy interventions that recognize the current reality in which the traditional social support system has been substantially degraded, necessitating the mainstreaming of formal psychosocial support for PLWHA. This proposal is also premised on results from the same qualitative substudy that informs this paper, which have been published elsewhere [52], pointing to the feasibility and acceptability of integrating depression management into routine HIV care. The mainstreaming of formal psychosocial support is anticipated to address some of the common challenges in delivery of mental health services in low-resource settings, including low demand for these services, which, from the qualitative substudy referred to above, was found to be in part a function of patients' lack of awareness about their mental illness, particularly depression. Mainstreaming of formal psychosocial support might also contribute to addressing the endemic shortage of mental health professionals in Uganda [53, 54] particularly if this engenders the training and leveraging of the existing healthcare workforce that is already providing HIV care to the same patients, within a new framework of integrated depression and HIV services. It will be noted that while the current HIV prevention and treatment guidelines in Uganda provide for integration of depression management into routine HIV care, the mechanics of achieving this are still in development. Indeed, through our pioneering HIV + D trial, we have established that such provision of integrated care may not after all result in increased burden of care on the part of health workers. For example, we note that depression management can boost HIV patients' CD4 count thus reducing viral load, and that this may lower the need for frequent visits to health facilities, consequently cutting down on health workers' work load. We also note that with relevant training targeted at the cadre of health workers that have traditionally provided HIV care, integration may benefit the patient by facilitating provision of different services by the same cadre of health workers on a single visit thereby minimising the frequency with which patients visit health care facilities in search of different specialised services.

Additionally, we propose a shift from individual to family-focused counselling programmes that would target not only the person living with HIV but also their family members. We recognise that for these family-focused counselling programmes to be of optimal benefit to PLWHA, they would necessarily also incorporate promotion of HIV status disclosure. This is in light of our findings suggesting that the benefits of social support would accrue when family members are sensitised but also when they (especially partners) are informed about the HIV status of the family member, to begin with. Accordingly, we propose that family-focused interventions also promote mutual disclosure among partners, and that delivery of such interventions be undertaken with keen consideration of the potential for partner fallout in case of discordance. Relatedly, we recognise the potential contribution of traditional and faith healers in mental health care as highlighted by several studies across Africa [55–57]. Nonetheless, further research within our study population exploring these alternative health care services is still deemed necessary for the purpose of establishing contextual suitability.

## 5. Conclusions

Social support from family members played a dominant role both directly and indirectly in influencing depression risk among persons living with HIV and AIDS. While previous studies have commonly depicted poverty as an independent risk factor for depression, our study finds that the influence of poverty was largely moderated by social (emotional) support from family members. This implied that in such cases, social support within family was considered by PLWHAs to be an important cushion against poverty as a cause of depression among persons living with HIV/AIDS. We propose the mainstreaming of formal psychosocial support and a shift from individual to family-focused counselling that would target both persons living with HIV and their family members.

## 6. Limitations of the Study

A limitation of our study was that data were collected from only four HIV clinics in one district in the central region of Uganda. Thus, while the inferences we draw from analysis of these data may be generalizable to the experience of depression among HIV patients in Uganda, especially considering the minimal variation in the country's sociocultural and economic conditions, we caution that these results may not fully depict HIV patients' experiences and perspectives on depression across the country.

## Figures and Tables

**Table 1:** Main topics covered and number of participants involved in the interviews with the different categories of participants at different time points.

Participant category	Number of participants	Topics covered during interview or focus group discussion (FGD)
PLWHA	11	At baseline: depression awareness, depression treatment experiences, and feasibility of integration of depression management into routine HIV care
	11	At midline: experiences with those delivering the treatment, challenges during treatment, and recommendations for change
	11	At endline: changes in health since the last therapy session, challenges encountered, what worked/did not work well, and recommendations for change

Adherent and nonadherent HIV patients (a subset of PLWHA who had not been interviewed) participating in exit interviews	11 adherent	Experiences and reasons for adherence/nonadherence
	6 nonadherent	

Lay health workers	1 FGD (with 8 participants)	At baseline: perspectives on depression and psychotherapy, the process of delivery of psychotherapy, and feasibility of integration
	1 FGD (with 8 participants)	At midline: experiences and challenges of delivering psychotherapy and recommendations for improving the treatment, ease of applying decision support algorithm
	1 FGD (with 8 participants)	At endline: experiences of managing depression and with patients, challenges of managing depression and perspectives on integration, ease of applying decision support algorithm

## Data Availability

The MRC/UVRI and LSHTM Uganda Research Unit operates an open data access and has a data sharing policy accessible at https://web.archive.org/web/20201201165922/http://www.mrcuganda.org/sites/default/files/publications/MRC_UVRI_Data_sharing_policy_December2015.pdf. The policy summarizes the conditions under which data collected by the Unit can be made available to other bona fide researchers, the way in which such researchers can apply to have access to the data and how data will be made available if an application for data sharing is approved. Should any other researchers need to have access to the data from which this manuscript was generated, the processes to access the data are well laid out in the policy. The corresponding and other co-author emails have been provided and they could be contacted anytime for further clarifications and/or support to access the data.

## Abbreviations

HIV/AIDS: Human immune deficiency virus/acquired immune deficiency syndrome or acquired immunodeficiency syndrome
ART: Antiretroviral therapy
PLWHA: Persons living with HIV and AIDS
HIV + D: Integrating the management of depression into routine HIV care in Uganda.
